# Deciphering functional diversity and structural determinants of substrate specificity in fungal glycoside hydrolase family 5_5 cellulases

**DOI:** 10.1128/aem.00417-26

**Published:** 2026-04-21

**Authors:** Jie Zheng, Ruiju Miao, Jianxin Zhang, Fei Zheng, Hanqing Liu, Xiao Wang, Xiaolu Wang, Huoqing Huang, Bin Yao, Jian Tian, Huiying Luo, Xing Qin

**Affiliations:** 1 State Key Laboratory of Animal Nutrition and Feeding, Institute of Animal Science, Chinese Academy of Agricultural Sciences 12661, Beijing, People's Republic of China; 2 Institute of Biotechnology and Health, Beijing Academy of Science and Technology 145003, Beijing, People's Republic of China; 3 College of Life Sciences, Inner Mongolia University 12576 , Hohhot, People's Republic of China; 4 China Academy of Urban Planning and Design 66533, Beijing, People's Republic of China; 5 College of Biological Sciences and Biotechnology, Beijing Forestry University 12380, Beijing, People's Republic of China; Shanghai Jiao Tong University, Shanghai, China

**Keywords:** hidden Markov models, glycoside hydrolase family 5_5, cellulase, mannanase, evolution

## Abstract

Fungal enzymes in glycoside hydrolase family 5 subfamily 5 (GH5_5) display notable catalytic diversity, efficiently degrading cellulose and sometimes mannan. However, the structural determinants and molecular mechanisms governing substrate preference in this enzyme family remain unclear. In this study, GH5_5 enzymes from fungi were systematically classified using profile-based sequence models and functionally characterized. Saturation mutagenesis combined with high-resolution crystal structure analysis of the bifunctional enzyme *Bs*Cel5B, exhibiting cellulase (CEL) activity of 941 ± 17 U/mg and mannanase (MAN) activity of 1,736 ± 34 U/mg, was employed to identify key residues controlling substrate specificity. Residue T100 in *Bs*Cel5B was identified as a major structural contributor associated with significant shifts in substrate preference. The T100V and T100N mutations resulted in 2.0-fold increases in MAN activity and 2.5-fold increases in CEL activity, respectively, generating bifunctional enzymes with enhanced substrate-specific activities. Similar substrate specificity trends were observed in several GH5_5 cellulase mutants. Their structural analysis indicated that substrate preference in fungal GH5_5 enzymes might be shaped by residual network-mediated alterations of the active-site geometry, with T100 acting as a second-shell regulatory element within a cooperative residue network. Together, these findings suggest a mechanistic framework for engineering catalytic specificity in GH5_5 enzymes.

**IMPORTANCE:**

Cellulose and mannan are major components of plant biomass, and enzymes capable of efficiently breaking them down are essential for sustainable biofuel production and biomass utilization. Fungal enzymes in GH5_5 are widely used for these purposes, yet their functional diversity has been difficult to predict or control. Substrate preference in these enzymes can be modulated by altering a single amino acid, offering a promising approach for tuning enzyme activity. The identification of a key residue that influences the balance between cellulose and mannan degradation provides valuable insights for engineering enzymes with tailored functions. These findings contribute to a deeper understanding of fungal biomass-degrading enzymes and support the rational design of more efficient catalysts for industrial and environmental applications.

## INTRODUCTION

Non-starch polysaccharides (NSPs), such as glucans, xylans, and mannans, are abundant in plant-derived feedstocks and lignocellulosic biomass. In animal diets, these polysaccharides act as anti-nutritional factors by increasing intestinal viscosity and impairing nutrient absorption (1). The synergistic action of cellulases (CELs) and mannanases (MANs) is crucial for NSP degradation. However, mannans and their hydrolysis products strongly inhibit CEL activity, thereby limiting overall saccharification efficiency (2, 3). Therefore, engineering glycoside hydrolases (GHs) with broad substrate specificity represents a promising strategy to overcome these challenges, potentially improving both animal nutrition and biomass-to-bioenergy conversion (4, 5).

Among glycoside hydrolases, the GH5 family represents one of the most substrate-diverse CEL groups (4), and the GH5_5 subfamily contains several bifunctional enzymes with both CEL and MAN activities (6–8). Nevertheless, the molecular basis of substrate promiscuity in GH5_5 enzymes remains unclear. Accumulating evidence suggests that subtle mutations at key active-site residues can modulate the recognition of structurally similar polysaccharides (9, 10), yet systematic identification of these determinants is still lacking.

To further illustrate the complexity of substrate recognition, structural differences between glucose and mannose must be considered. Glucose and mannose differ only at the C2 hydroxyl group: glucose has an equatorial orientation, whereas mannose has an axial orientation (11). Despite this subtle structural difference, the impact on enzymatic recognition and catalytic efficiency can be substantial. Although some studies have investigated the catalytic mechanisms of CELs acting on mannan substrates, a comprehensive molecular understanding is still lacking. For example, the GH5_25 enzyme *Tm*Cel5A from *Thermotoga maritima* exhibits dual CEL and MAN activities, and structural analyses revealed minimal global conformational changes upon substrate binding (12, 13). A flexible loop near the active site has been proposed to contribute to catalytic adaptability (14). However, how such local structural features translate into substrate selectivity is not fully understood. Elucidating how CELs recognize and catalyze such structurally similar substrates is therefore a key challenge for understanding substrate promiscuity and for guiding rational enzyme engineering. These gaps highlight the need for a systematic investigation of GH5_5 CELs. Given the scale and complexity of sequence-activity relationships, artificial intelligence-assisted statistical modeling offers a powerful framework for uncovering hidden sequence-function patterns (15).

This study aims to elucidate the molecular determinants of substrate promiscuity in GH5_5 CELs. To this end, a hidden Markov model (HMM)-based approach was employed to screen and classify fungal GH5_5 members. Bioinformatic analysis was further integrated with mutagenesis and structural biology to investigate the key factors underlying functional divergence. Through this integrative strategy, new insights are provided into how the examined GH5_5 enzymes accommodate multiple substrates. Ultimately, this work provides a conceptual and methodological framework for the rational engineering of multifunctional GHs, with potential implications for both fundamental enzymology and the development of cost-effective solutions in feed utilization and biomass-to-bioenergy conversion.

## RESULTS

### Functional classification of GH5_5 CELs

To elucidate the functional diversity of fungal GH5_5 CELs, 25 representative enzymes from different fungal species were selected (Table S1). Using carboxymethyl cellulose sodium (CMC-Na) and locust bean gum (LBG) as the respective glucan and mannan substrates, the optimal reaction conditions of each enzyme were determined. Overall, these enzymes exhibited maximal activity under mildly acidic conditions and at moderate-to-high temperatures (Table S2). Notably, for each enzyme, the optimal pH and optimal temperature were identical when assayed with either substrate.

**Fig 1:**
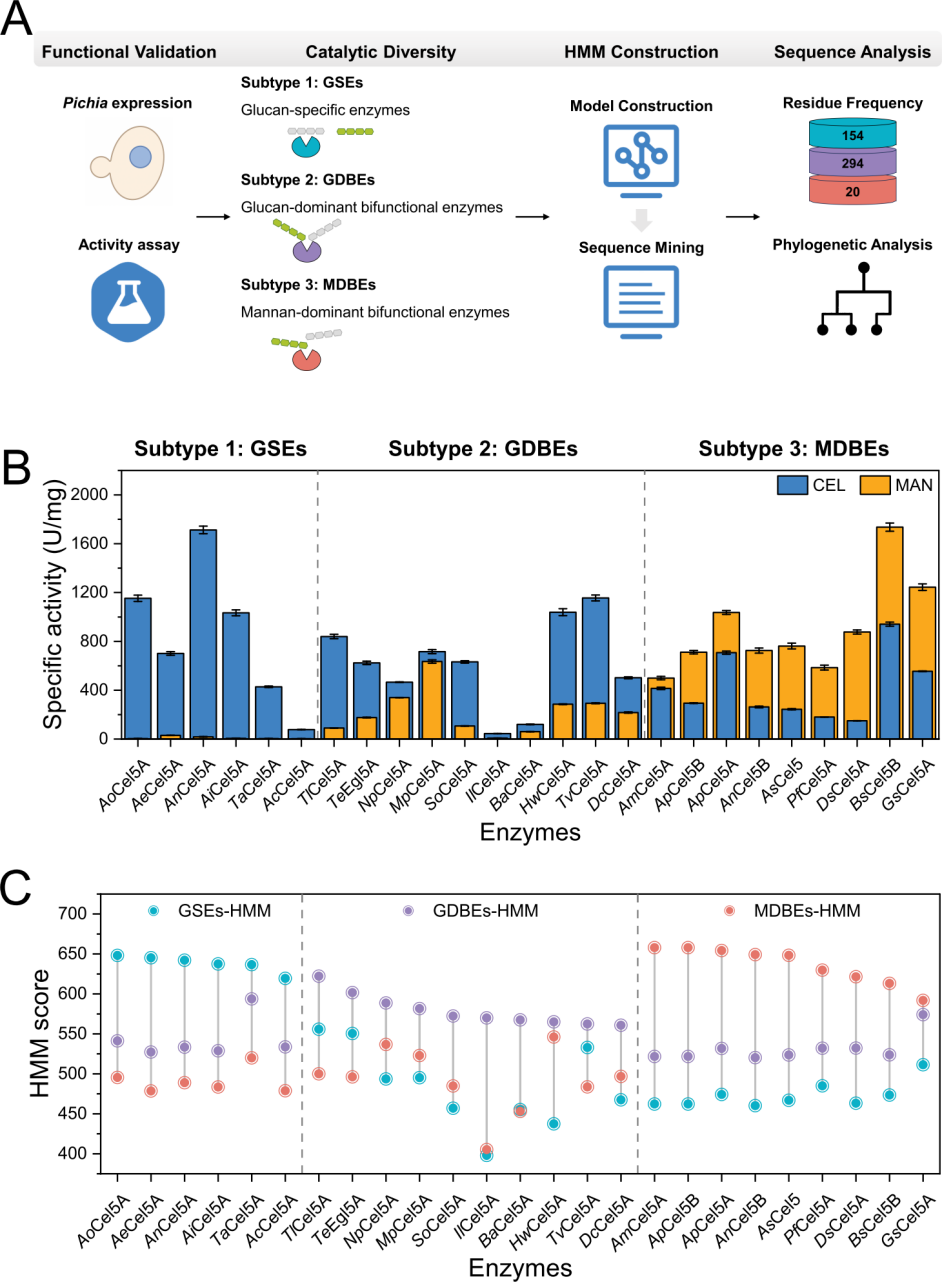
Functional classification leading to sequence mining of fungal GH5_5 CELs. (**A**) Stepwise analytical workflow involving HMM-guided classification and downstream sequence mining. (**B**) Catalytic activity assays of CEL and MAN for 25 candidates. The blue bars represent the catalytic activity of CEL, while the orange bars represent the catalytic activity of MAN. Each bar represents the mean of three technical replicates, and the error bars indicate the standard deviation. (**C**) HMM score analysis of 25 candidates.

Based on activities measured under their respective optimal reaction conditions, the enzymes were classified into three functional groups. Six enzymes, including *An*Cel5A, demonstrated activity that was specific to glucan. In contrast, ten enzymes, such as *Hw*Cel5A, were able to act on both substrates but showed a higher level of activity on glucan. Additionally, nine enzymes, exemplified by *Bs*Cel5B, were active on both substrates but displayed greater activity toward mannan (Fig. 1A and B). Notably, *Bs*Cel5B from *Bispora* sp. MEY-1 showed MAN activity of 1,736 ± 34 U/mg and CEL activity of 941 ± 17 U/mg. *Ds*Cel5A from *Dothistroma septosporum* showed MAN activity of 877 ± 17 U/mg, which was 5.84-fold higher than its CEL activity. Both enzymes showed a clear preference for mannan, forming a previously uncharacterized subclass within the examined enzyme set.

For systematic subtyping, the CEL-to-MAN activity ratio (CEL/MAN) was used as a quantitative index. Enzymes with ratios greater than 10 were designated as glucan-specific enzymes (GSEs). Enzymes with ratios between 1 and 10 were classified as glucan-dominant bifunctional enzymes (GDBEs), and those with ratios less than 1 were classified as mannan-dominant bifunctional enzymes (MDBEs). In this study, MDBEs were identified that showed stronger mannan preference and had not been reported previously for GH5_5 enzymes, with mannan activity exceeding CEL activity. This functional classification highlighted the catalytic divergence observed among the selected GH5_5 enzymes and provided a framework for subsequent sequence mining.

### Classification leading to sequence mining of GH5_5 CELs

To systematically classify and mine GH5_5 CELs, three HMM models (15, 16) were constructed using 25 experimentally validated enzymes (Fig. 1A and C). These models corresponded to the three subtypes: GSEs-HMM, GDBEs-HMM, and MDBEs-HMM. Scoring results showed strong consistency with the experimentally determined enzymatic activity profiles. GSEs, GDBEs, and MDBEs all achieved the highest scores in their respective models (Fig. 1C), further validating the reliability of the constructed HMM classifiers. The results also showed that the enzymes of the three subtypes exhibited distinct sequence features within the analyzed data set. To further evaluate model robustness, leave-one-out (LOO) cross-validation was performed for the three subtype-specific HMM models (Table S3). The overall LOO classification accuracy was 84%, indicating a high level of subtype classification consistency across the data set.

From the UniRef90 database, a total of 468 fungal GH5_5 CEL sequences were retrieved using the three HMM models, after removing redundant entries and filtering for sequence lengths of 250–500 amino acids (Fig. S1). Based on HMM scores, the sequences were functionally classified into 154 GSEs, 294 GDBEs, and 20 MDBEs. Notably, bifunctional sequences (GDBEs + MDBEs) accounted for 67% of the total, highlighting the prevalent representation of predicted dual-substrate catalytic capacity among the retrieved sequences. Phylogenetic analysis of the 468 sequences revealed clear subtype-specific clustering. In particular, MDBEs formed a highly cohesive evolutionary clade (Fig. S1), providing support for the association between functional specialization and evolutionary divergence.

### Identification of key residues associated with catalytic diversity

**Fig 2:**
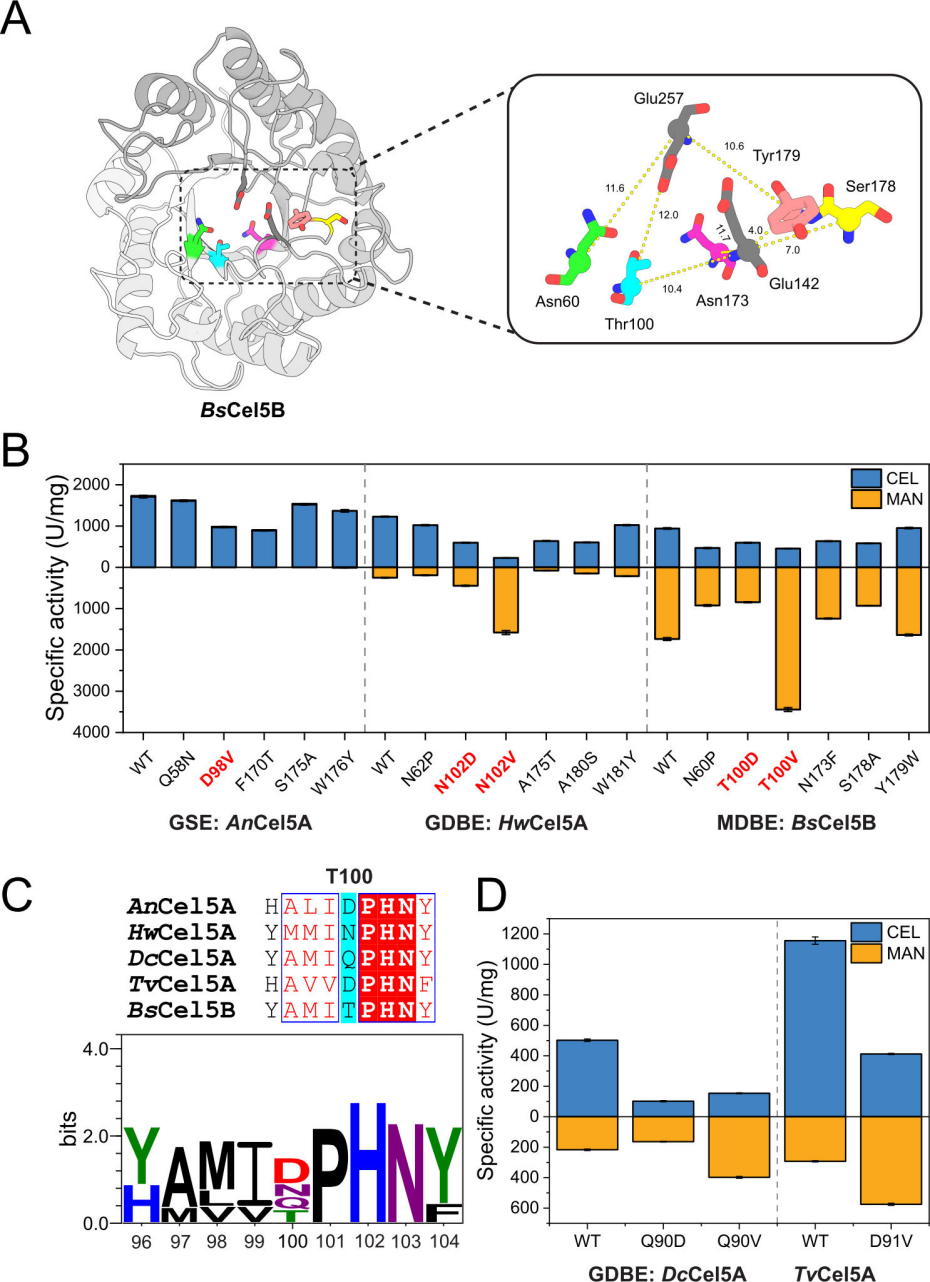
Analysis of key residues influencing substrate specificity in GH5_5 enzymes. (**A**) Cα distances between substrate-specific residues with significant frequency differences and the catalytic center. (**B**) Effects of key residue mutations on substrate preference in GSE, GDBE, and MDBE subtype enzymes. Bars represent the mean of three technical replicates, with error bars indicating the standard deviation. The blue bars correspond to CEL activity, and the orange bars correspond to MAN activity. (**C**) Sequence alignment of the T100-equivalent position among representative enzymes from the GSE, GDBE, and MDBE subtypes. (**D**) Catalytic activity assays of site-directed mutants at the T100-equivalent position in *Dc*Cel5A and *Tv*Cel5A. The blue and orange bars represent CEL and MAN activity, respectively, with each bar showing the mean of three technical replicates and error bars indicating standard deviation.

The structural distinction between glucose and mannose at the C2 hydroxyl group suggests that substrate recognition is mediated by residues near the catalytic center. To identify amino acid residues potentially involved in substrate recognition, residue conservation and frequency distribution analyses were performed across the three functional subtypes of GH5_5 enzymes. A total of 26 positions with significant differences in residue frequencies among the subtypes were identified (Table S4). These subtype-dependent frequency variations formed the basis for candidate residue selection. Structural distance analysis was then performed to refine candidates based on spatial proximity to the catalytic center, as summarized in Table S5. Only five of these frequency-variable residues were located within 12 Å of the catalytic center, as determined by Cα distance (Fig. 2A; Table S5). This limited set of residues is likely to represent important contributors to substrate preference.

For the evaluation of the functional relevance of these five residues in catalytic diversity, three representative enzymes were selected from the three subtypes. At each target site, the native residue was replaced with the predominant residue found in the alternative subtypes. Overall, mutations at these key positions caused marked shifts in catalytic activity and substrate preference. In *Bs*Cel5B, the T100V mutant nearly doubled MAN activity (from 1,736 ± 34 to 3,445 ± 47 U/mg), whereas T100D sharply reduced it. In *An*Cel5A, D98V lowered CEL activity by ~40%. In *Hw*Cel5A, N102V decreased CEL activity (from 1,227 ± 12 to 229 ± 1 U/mg) but enhanced MAN activity more than sixfold (from 251 ± 9 to 1,580 ± 47 U/mg), shifting substrate preference toward mannan (Fig. 2B). Collectively, these results demonstrate that Thr100 in *Bs*Cel5B, together with analogous positions in *An*Cel5A and *Hw*Cel5A, plays a pivotal role in modulating substrate preference. This effect likely arises from their involvement in substrate recognition and binding, thereby contributing to the functional divergence between CEL and MAN activities observed in the examined GH5_5 enzymes.

### Validation of the conserved regulatory role of T100 in substrate preference

To test whether the regulatory function of T100 is shared among representative GH5_5 enzymes, two additional representative GDBE-subtype enzymes were selected for site-directed mutagenesis at the T100-equivalent positions. Amino acid frequency analysis showed that the T100 site in *Bs*Cel5B exhibits subtype-dependent conservation: Asp is predominantly present in GSEs and GDBEs, whereas Val is more common in MDBEs (Fig. 2C; Table S4). This subtype-specific frequency pattern further supports the functional relevance of this position in substrate differentiation. Functional assays yielded consistent results across the selected enzymes. In GDBE-subtype enzymes, Q90V in *Dc*Cel5A and D91V in *Tv*Cel5A increased MAN activity by 83% and 96%, respectively, while reducing CEL activity (Fig. 2D).

These results underscore the important contribution of the T100-equivalent residue to influencing substrate preference in the examined GH5_5 enzymes. In the tested enzymes, precise substitution at this site enabled a shift between GDBE- and MDBE-like activity profiles while preserving dual catalytic activity. This pattern is consistent with the observed subtype-associated residue distributions.

### Saturation mutagenesis reveals the negative trade-off mechanism

**Fig 3:**
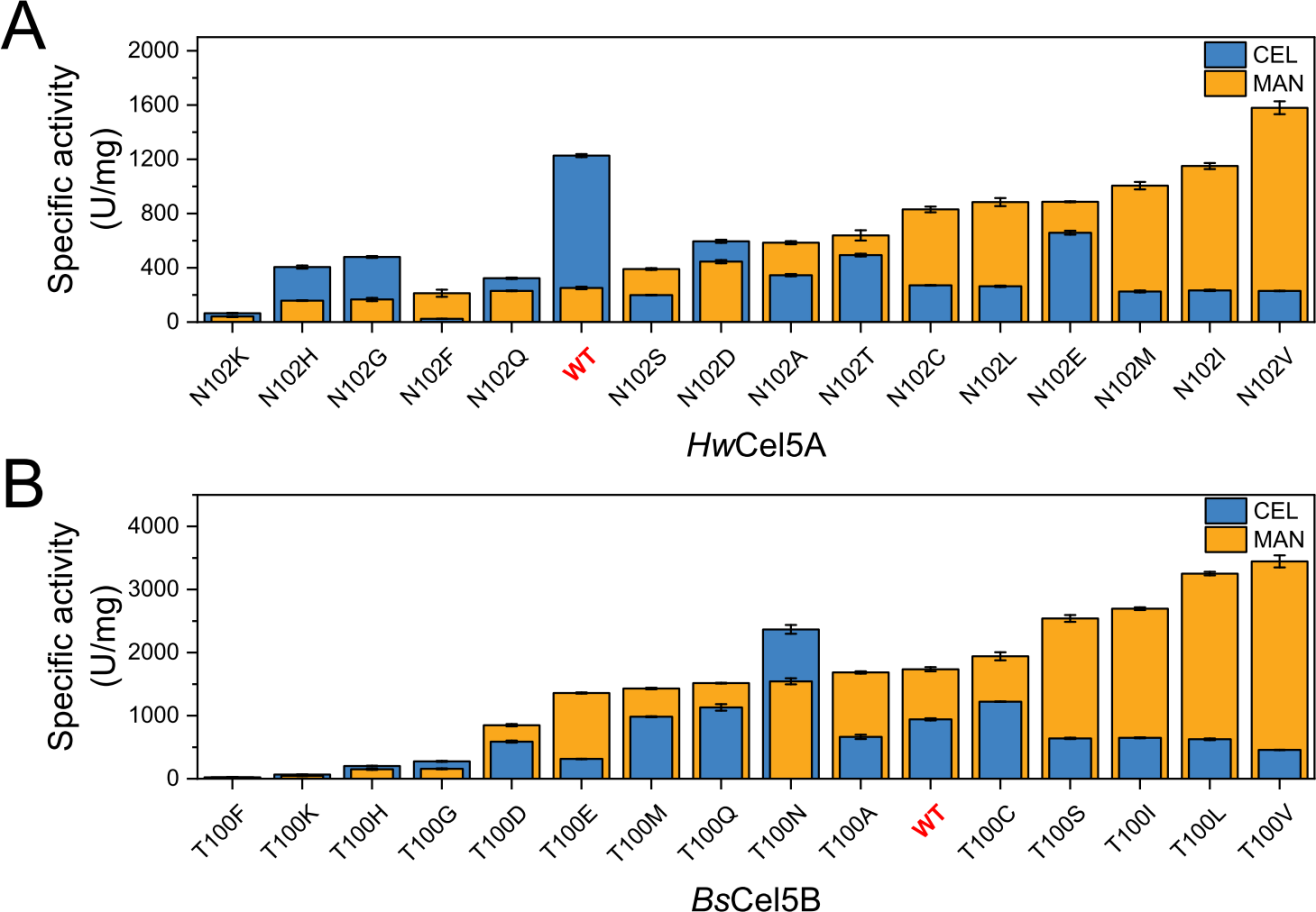
Saturation mutagenesis analysis of the T100 site. (**A and B**) Catalytic activity assays of saturation mutants at the T100-equivalent position in *Hw*Cel5A and *Bs*Cel5B. Each bar represents the average of three technical replicates, with error bars showing the standard deviation. The blue bars correspond to CEL activity, and the orange bars correspond to MAN activity.

To further investigate the functional role of residue T100 in substrate selection, saturation mutagenesis was performed at this site. *Hw*Cel5A and *Bs*Cel5B were selected as representative enzymes of the GDBE and MDBE subtypes, respectively (Fig. 3A and B). Mutations T100Y, T100W, T100P, and T100R in *Bs*Cel5B completely abolished catalytic activity. A similar loss of activity was observed for the corresponding substitutions (Y/W/P/R) at the N102 position in *Hw*Cel5A. These results indicated that this position was essential for maintaining structural stability or catalytic function in both GDBE- and MDBE-subtype enzymes.

Among the non-lethal variants, the N102V mutation in *Hw*Cel5A led to a 5.42-fold increase in catalytic efficiency (*k*_cat_/*K*_m_) toward mannan. At the same time, CEL activity decreased to 0.26-fold of the wild-type level. This shift converted the enzyme’s substrate preference from the GDBE to the MDBE subtype (Fig. 3A; Table 1). A similar shift in substrate preference was observed in *Bs*Cel5B upon mutation at the T100 position. Among the *Bs*Cel5B variants, T100V showed the highest catalytic efficiency toward mannan. Its *k*_cat_/*K*_m_ increased by 1.27-fold, while CEL activity decreased to 0.66-fold of the wild-type level (Fig. 3B; Table 1). Replacement of T100 with hydrophobic residues (Val, Leu, Ile) or polar residues (Ser, Thr, Cys) generally shifted enzyme activity toward MAN. These results suggest that the T100V mutation causes a reciprocal change in CEL and MAN activities, simultaneously enhancing MAN activity in the MDBE background and inducing MDBE-like behavior in GDBE-subtype enzymes.

**TABLE 1:** Kinetic parameters of *Hw*Cel5A and *Bs*Cel5B^*a*^

Enzyme	*k*_cat_/*K*_m_ (S^−1^M^−1^)	
	CMC-Na	LBG
*Hw*Cel5A (WT)	96 ± 10	139 ± 18
*Hw*Cel5A (N102V)	25 ± 7	754 ± 65
*Bs*Cel5B (WT)	122 ± 7	613 ± 34
*Bs*Cel5B (T100N)	637 ± 5	379 ± 34
*Bs*Cel5B (T100V)	80 ± 6	779 ± 51

^*a*^ All samples were analyzed in triplicate in each independent experiment, and the mean and standard deviation were calculated.

In contrast to the increase in MAN activity caused by T100V, the T100N mutation in *Bs*Cel5B markedly increased CEL activity by 5.21-fold, while simultaneously reducing MAN activity to 0.62-fold of the wild-type level (Fig. 3B; Table 1). This substitution reversed the enzyme’s substrate preference, resulting in a functional shift from MDBE-like to GDBE-like behavior. Similar inverse trade-offs between CEL and MAN activities were consistently observed in the examined GH5_5 enzymes. Enhancement of activity toward one substrate was often accompanied by a proportional reduction in the other, reflecting a tightly coupled substrate preference mechanism. These reciprocal activity patterns likely arise from intrinsic evolutionary constraints that favor functional specialization. Notably, this trade-off can be strategically rewired through precise substitutions at key positions, such as T100, thereby providing a potential approach for modulating substrate specificity in GH5_5 CELs.

### Structural elucidation of *Bs*Cel5B by X-ray crystallography

**Fig 4:**
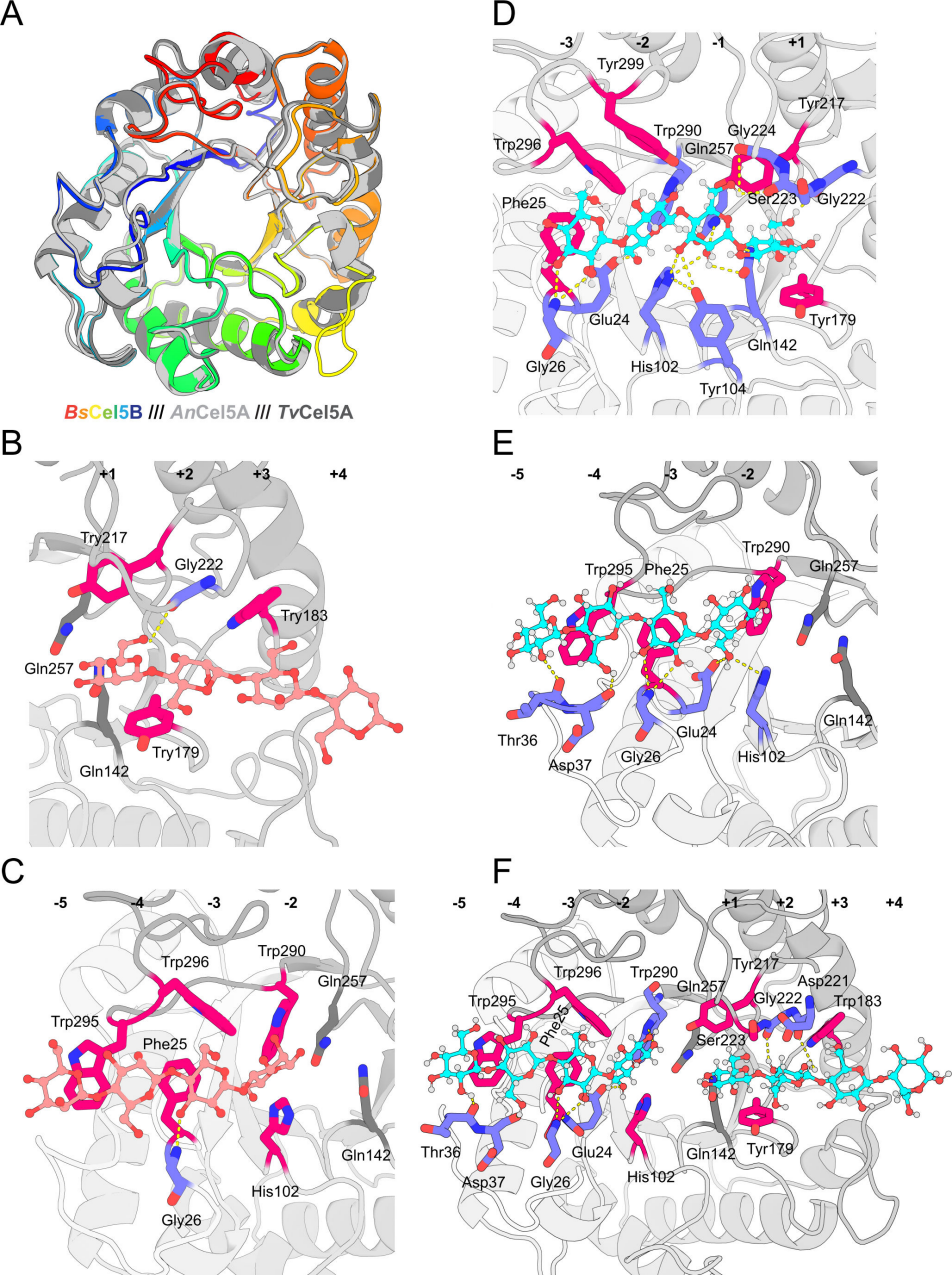
Crystal structure analysis of *Bs*Cel5B. (**A**) Structural comparison of *Bs*Cel5B (PDB ID: 8ZLG), *An*Cel5A (PDB ID: 5I79), and *Tv*Cel5A (PDB ID: 5L9C). *Bs*Cel5B is shown in rainbow colors, while *An*Cel5A and *Tv*Cel5A are displayed in light gray and dark gray, respectively. (**B and C**) Crystal structure of *Bs*Cel5B-E142Q/E257Q in complex with MTT. (**D–F**) Crystal structure of *Bs*Cel5B-E142Q/E257Q in complex with CTT. Catalytic site mutations E142Q and E257Q are shown in dark gray. Residues involved in hydrophobic interactions with the substrate are highlighted in hot pink, and those forming hydrogen bonds are shown in slate.

To gain structural insights into the catalytic diversity of GH5_5 enzymes, X-ray crystal structures were determined for both wild-type *Bs*Cel5B and the catalytically inactive double mutant *Bs*Cel5B-E142Q/E257Q. Both apo forms and substrate-bound complexes with cellotetraose (CTT) and mannotetraose (MTT) were solved (Table S6). *Bs*Cel5B adopts the canonical TIM-barrel fold characteristic of the GH5 family. Structural alignment with two other GH5_5 representatives, *An*Cel5A (GSE subtype, PDB: 5I79) and *Tv*Cel5A (GDBE subtype, PDB: 5L9C), yielded low backbone root-mean-square deviations (0.461 and 0.476 Å, respectively), indicating a high degree of structural conservation across functional subtypes (Fig. 4A). This conserved fold likely facilitates the recognition of multiple substrates with subtle stereochemical differences, such as glucan and mannan, which differ only at the C2 hydroxyl position.

Substrate binding modes were further characterized using the *Bs*Cel5B-E142Q/E257Q mutant. In both the MTT- and CTT-bound complexes, four polypeptide chains were present in the asymmetric unit, showing variable substrate occupancies. The detailed binding patterns of MTT and CTT are illustrated in Fig. 4B, C, and Fig. 4D through F, respectively. These results demonstrate that *Bs*Cel5B can accommodate at least two tetrasaccharide molecules simultaneously within its binding cleft.

To dissect the molecular basis of substrate recognition and catalysis, detailed interactions were examined in the *Bs*Cel5B-E142Q/E257Q complexes. The substrate-binding cleft features several aromatic residues (Phe25, Tyr179, Trp290, Trp295, Trp296, and Tyr299) that engage in π–π and σ–π stacking interactions with the sugar rings, thereby enhancing carbohydrate affinity and specificity. In the catalytically inactive double mutant, the two catalytic glutamates are replaced by glutamines, allowing capture of a stable pre-catalytic complex. At the catalytic center, the amide group of Gln142 forms a hydrogen bond (N···O = 3.2 Å) with the bridging oxygen between the −1 and +1 glucosyl units, mimicking the protonation step normally mediated by Glu142 in the wild-type enzyme. Gln257 is positioned 3.7 Å from the C1 atom of the −1 sugar in CTT, corresponding to the nucleophilic attack site where a covalent glycosyl-enzyme intermediate would typically form (Fig. 4D). In addition to these catalytic-site interactions, His102 and Tyr104 form stabilizing hydrogen bonds with the O2 and O3 atoms of the −1 sugar, thereby contributing to precise substrate positioning (Fig. 4D). Collectively, the four residues Gln142, Gln257, His102, and Tyr104 appear to lock the −1 sugar into a distorted boat-like conformation, a feature not previously reported among GH5_5 fungal CELs.

### Conformational variations of key residues determine substrate preference

**Fig 5:**
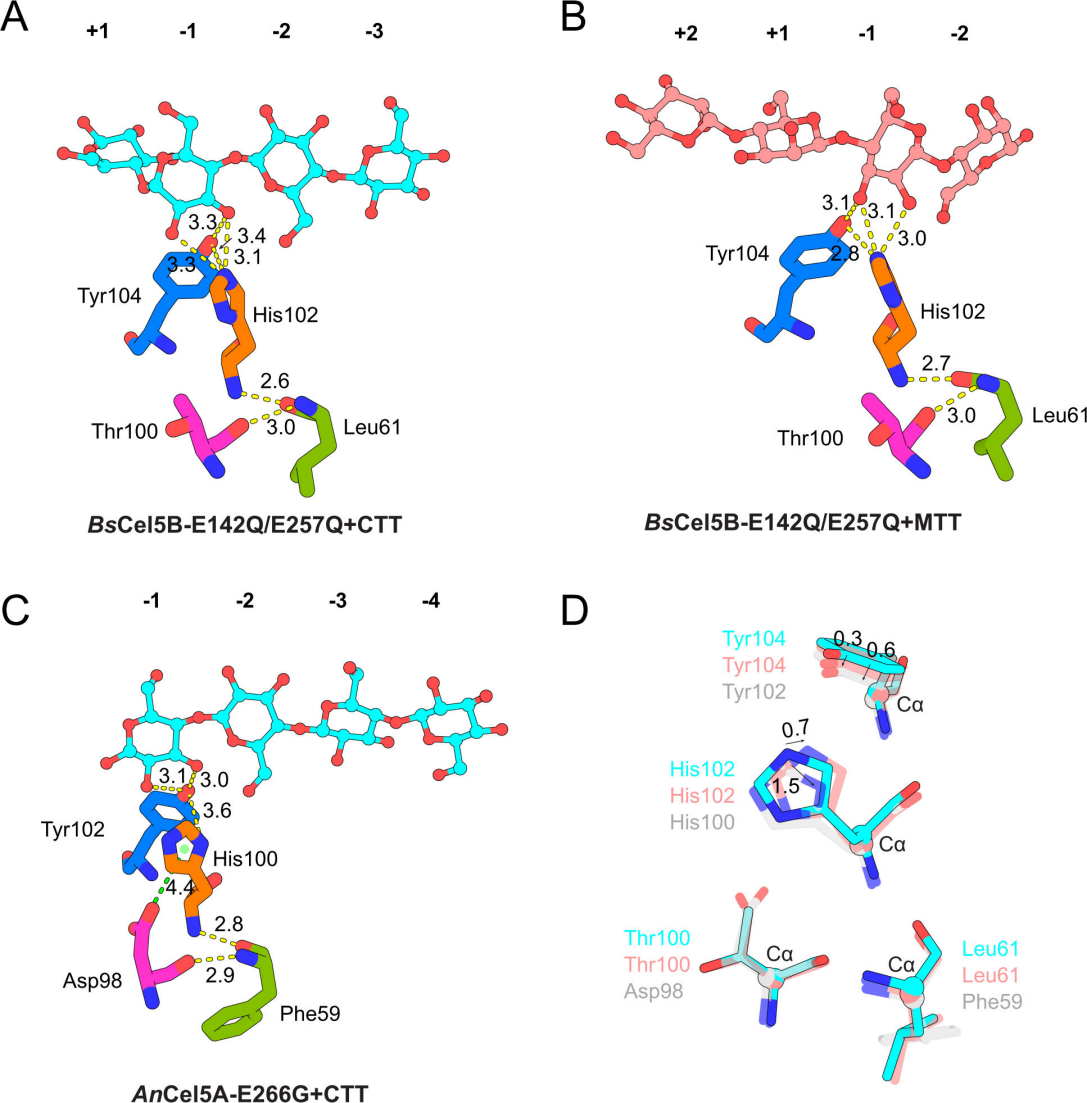
Analysis of key substrate-binding residues at the −1 subsite. (**A–C**) Conformational changes of −1 subsite residues in catalytically inactive mutants of *Bs*Cel5B and *An*Cel5A upon binding to CTT and MTT substrates. (**D**) Structural comparison of key −1 subsite residues in *Bs*Cel5B and *An*Cel5A mutants bound to CTT and MTT. Residues in *Bs*Cel5B bound to CTT are shown in cyan, those bound to MTT in pink, and those in *An*Cel5A bound to CTT in light gray.

Although His102 and Tyr104 are conserved across GH5_5 enzymes and form similar hydrogen bonding patterns with both glucan and mannan substrates (Fig. 2C and 5A through C), differences in binding affinity suggest a more nuanced structural basis for substrate discrimination (Fig. 2B). In particular, structural snapshots of the *Bs*Cel5B–MTT complex revealed partial occupancy within the active site, with unresolved interactions spanning the −1 to +1 subsites (17). To further clarify the role of these conserved residues in catalytic diversity and substrate selectivity, an MTT molecule was modeled into the −2 to +2 subsites of the *Bs*Cel5B-E142Q/E257Q+MTT complex (Fig. 5B), and the resulting enzyme–substrate interactions were examined.

Previous studies have demonstrated that the −1 subsite plays a pivotal role in substrate binding and enzymatic activity across the GH5 family, and this region is highly conserved among its members (18). In *Bs*Cel5B, the −1 subsite residues His102 and Tyr104 engage in hydrogen bonding interactions with the substrate. Specifically, His102 forms two hydrogen bonds with the C2–OH and C3–OH groups of both CTT and MTT, whereas Tyr104 contributes a single hydrogen bond to the C3–OH group. Importantly, the identity and number of these hydrogen bonds remain unchanged regardless of whether *Bs*Cel5B is bound to CTT or MTT (Fig. 5A and B), highlighting the enzyme’s capacity to recognize and catalyze both substrates. Despite this conservation, MTT binding results in shorter hydrogen bond distances compared to CTT binding (Fig. 5A and B), indicating stronger interactions that correlate well with kinetic data. This enhanced affinity likely arises from the axial configuration of the C2–OH group in mannan, together with subtle conformational adjustments of His102 and Tyr104. The crystal structure of *An*Cel5A (PDB: 5I79, GSE subtype) in complex with CTT provides a comparative framework for examining substrate specificity between glucan and mannan. Structural overlay showed that MTT binding in *Bs*Cel5B caused modest shifts in His102 and Tyr104, whereas CTT binding in *An*Cel5A induced more pronounced displacements of the corresponding residues (Fig. 5D). These conformational changes are likely linked to differences in substrate specificity and catalytic efficiency between the two enzymes.

Further structural analysis suggests that this differential behavior is modulated by residue T100. Although T100 does not directly interact with the substrate, it participates in a hydrogen-bonding network involving His102, Leu61, and Tyr104 that influences the local conformation of the active site. In *An*Cel5A, Asp98 forms an ionic interaction with His100, which in turn weakens the hydrogen bond between His100 and Tyr102 (Fig. 5C). By contrast, *Bs*Cel5B harbors a polar but uncharged T100 at the equivalent position. The absence of electrostatic attraction with His102 allows for a more stable hydrogen bond between His102 and Tyr104, thereby enhancing MTT-binding affinity (Fig. 5A and B). Moreover, substitution of T100 with hydrophobic residues, such as Val, Leu, or Ile, may increase the torsional angle of His102 through hydrophobic repulsion, thereby further stabilizing the His102–Tyr104 hydrogen bond.

Taken together, mutagenesis and structural analyses collectively indicate that T100 is an important structural contributor to substrate preference in the examined GH5_5 enzymes. Notably, T100 does not act through direct substrate contact. Instead, it participates in a local hydrogen-bonding network that modulates the conformational stability of the His102–Tyr104 region, thereby altering the accommodation of different substrates within the −1 subsite. In this context, T100 may be regarded as a regulatory hub within a cooperative residue network. Its effect depends on conformational coupling with neighboring residues rather than functioning as an independent determinant.

## DISCUSSION

In this study, bioinformatic tools were utilized in conjunction with a comprehensive data set of GH5_5 fungal cellulases to explore the determinants of substrate diversity within this enzyme subfamily. A previously unidentified subtype with predominant activity toward mannose was discovered. Through screening of candidate residues, a crucial site governing catalytic specificity was identified. Subsequent saturation mutagenesis, coupled with high-resolution crystallography, revealed a structural basis for a “negative trade-off” effect influencing substrate preference. These findings underscore the presence of a substrate preference network-mediated regulatory mechanism modulated by single-residue mutations, resulting in notable activity enhancements ranging from threefold to sixfold, representing some of the most significant improvements documented to date. Together, these outcomes offer fresh perspectives on substrate selectivity and provide insights into functional divergence within GH5_5 CELs, laying the groundwork for enzyme manipulation and the modification of substrate specificity.

To avoid the risk of overfitting, LOO cross-validation was performed. Despite the limited number of experimentally validated GSEs, GDBEs, and MDBEs, the LOO results support the robustness of the three subtype-specific HMM classifiers. The majority of sequences were consistently and stably assigned to their respective subtypes, indicating clear and stable discriminative boundaries. Additionally, the number of predicted MDBEs remained low (*n* = 20), and they formed a well-supported, phylogenetically coherent clade without excessive expansion (Fig. S1). These results suggest that the HMM classifiers capture subtype-specific features, rather than overfitting to a small data set.

To minimize the impact of experimental parameter variations on enzymatic reaction outcomes, the catalytic performance of 25 representative enzymes was evaluated under optimal reaction conditions for both cellulose and mannan substrates. Notably, these enzymes exhibited identical optimal pH and temperature for the degradation of both substrates (Table S2). This observation suggests that the catalytic processes for both substrates likely occur within similar active-site microenvironments and follow comparable key catalytic steps. Therefore, the observed differences in substrate preference are more likely attributable to subtle variations in substrate binding and positioning, rather than fundamental alterations in the overall catalytic mechanism.

Beyond sequence-based classification, structural analysis provides mechanistic insights into the observed functional divergence. Previous studies have attempted to reveal the structural basis of catalytic diversity through crystallographic analysis. Nevertheless, the minimal structural differences between glucan and mannan substrates make it difficult to identify the specific residues involved (13). While phylogenetic analyses have demonstrated that catalytic diversity is widespread across the GH5 family (19), their resolution may be insufficient to capture subtle structural and functional correlations. In this study, structural analysis revealed key substrate binding modes and catalytic residue interactions in the β-1,4-glycosidic bond hydrolysis process. Notably, an unusual distorted boat-like conformation of the −1 sugar was observed, which resembles the B_2,5_ transition-state mimic reported in α-mannanases. This conformation may lower the activation energy for glycosidic bond cleavage by stabilizing an oxocarbenium-ion-like geometry (15). Such a transition-state mimic structure may be a key factor in the substrate selectivity and catalytic efficiency diversity within the GH5_5 family of enzymes. Although this study provides experimental evidence for this transition-state conformation, further computational simulations and experimental data, particularly in the context of different substrates and mutants, will be essential to clarify the specific contribution of this conformation to glycosidic bond hydrolysis.

Enzyme evolution is inherently complex, and most evolutionary changes result in only subtle functional modulations, a process often referred to as “gradual evolution.” In contrast, the present study suggests that a single-point mutation at the second-shell residue T100 is capable of inducing dramatic shifts in substrate specificity. This effect is most plausibly mediated by alterations in the substrate-binding pocket or catalytic residues (20–22). In addition, both proximal residues that engage in direct interactions and distal residues that exert indirect long-range effects are likely to cooperatively modulate substrate binding and catalytic activity (23, 24). Nevertheless, the identification of network-critical residues and the elucidation of their contributions to biological adaptation remain considerable challenges (21, 25, 26).

Beyond the scope of the main investigation, residues adjacent to T100 were systematically substituted, and machine learning approaches were employed to identify functionally important distal residues in GSE-subtype enzymes. The resulting mutants exhibited a significant reduction in CEL activity. However, no corresponding increase in MAN activity was observed. These findings indicate that catalytic diversity is governed by highly position-specific mechanisms, despite the high overall sequence identity (~60%) among GH5_5 CELs. Although previous studies have successfully uncovered such distal determinants (19), the present analysis was constrained by the limited number of experimentally validated MDBEs (fewer than 30), which hindered the extraction of robust sequence features representative of this subtype.

### Conclusion

This study systematically investigated the mechanisms underlying catalytic diversity in GH5_5 CELs and elucidated their functional evolution at the molecular level. Key residues associated with catalytic diversity, along with their physicochemical properties and interaction networks, were identified. The results suggest that this diversity is intricately linked to the functional divergence of these enzymes, highlighting its significance in enzyme specialization. Notably, crucial residues can indirectly impact substrate preference and catalytic specificity through structural and dynamic networks. These insights into the structural and mechanistic characteristics of GH5_5 and its superfamily offer valuable guidance for enzyme screening and redesign in chemical, medical, and industrial sectors. Moreover, a deeper comprehension of catalytic diversity in CELs could aid in the development of sustainable bioeconomy strategies, particularly in agriculture, thereby informing both theoretical frameworks and practical implementations.

## MATERIALS AND METHODS

### Construction of hidden Markov models

HMMs were constructed from multiple sequence alignments (MSAs) using the local version of HMMER software (v3.3.1) (27). MSAs were generated using ClustalW for each of the functional subtypes. The resulting HMM profiles were used to query the UniRef90 protein database via the hmmsearch tool within the HMMER suite. Proteins that matched the corresponding HMM profiles with an E-value below 0.001 were retained, and only sequences with lengths below 500 amino acids were considered valid. To evaluate model robustness and mitigate potential overfitting, LOO cross-validation was performed for the three subtype-specific HMM models.

### Sequence data sets

The initial data set consisted of 25 sequences, which are listed in the supplemental material. HMMER searches were performed against the UniRef90 database using default parameters. Due to the high sequence diversity of GH5_5 CELs, only sequences with ≥60% query coverage were retained to exclude a large number of fragmentary hits returned by hmmsearch. In addition, a 99% sequence identity threshold was applied to remove redundant entries. Finally, a curated set of 468 fungal-derived CELs, each with approximately 300 amino acids and containing only the catalytic domain, was compiled for subsequent phylogenetic analysis.

### Creation of the phylogenetic tree

Multiple sequence alignments of the 468 fungal-derived CELs, obtained from the UniRef90 database by mining and screening, were generated using MAFFT (28). The resulting alignments were then used to construct a maximum-likelihood phylogenetic tree using FastTree with default parameters (29).

### Strains, plasmids, and chemicals

*Escherichia coli* Trans1-T1 (TransGen Biotech Co., Ltd., Beijing, China) and *Pichia pastoris* GS115 (Invitrogen, Carlsbad, CA, USA) were used as the cloning and expression hosts, respectively. The plasmid pPIC9 (Invitrogen) was employed for heterologous gene expression. Site-directed mutagenesis was performed using the Fast Mutagenesis System Kit (TransGen Biotech). The restriction enzyme *Bgl*II was purchased from Thermo Fisher Scientific (Waltham, MA, USA). CMC-Na (medium viscosity) and LBG were obtained from Sigma-Aldrich (St. Louis, MO, USA) and used as substrates. All chemicals were of analytical grade and commercially available. Endo-β-N-acetylglucosaminidase H (Endo H; New England Biolabs, Ipswich, MA, USA) was used to remove N-linked glycans. The concentrations of purified recombinant enzymes were determined using the Bradford protein assay.

### Gene cloning, expression, and site-directed mutagenesis

A total of 25 representative GH5_5 CEL sequences with known functional characteristics reported in the literature were codon-optimized and synthesized for expression in *Pichia pastoris*. All gene sequences (Table S1) were cloned without their native signal peptides into the pPIC9 vector. The constructs were linearized with *Bgl*II and subsequently electroporated into *Pichia pastoris* GS115 for expression. Mutant variants were generated using plasmids pPIC9-*Bs*Cel5B, pPIC9-*Hw*Cel5A, pPIC9-*An*Cel5A, pPIC9-*Dc*Cel5A, and pPIC9-*Tv*Cel5A as templates. Expression and purification of recombinant CELs were performed as previously described (30).

### Protein crystallization

Crystals of wild-type *Bs*Cel5B and the catalytically inactive mutant *Bs*Cel5B-E142Q/E257Q in complex with CTT or MTT were obtained at 18°C using the sitting-drop vapor diffusion method. Initial crystallization conditions consisted of mixing 1 μL of 20 mg/mL protein solution with 1 μL of reservoir solution containing 0.1 M Tris (pH 7.5), 0.2 M sodium acetate, and 32% (wt/vol) PEG 4000. Crystals typically formed within 5 days and were cryoprotected by transferring them into the reservoir solution supplemented with 15% (vol/vol) glycerol, followed by flash cooling in liquid nitrogen. The crystallization condition for the E142Q/E257Q mutant was identical to that of the wild-type protein. Complex crystals of *Bs*Cel5B-E142Q/E257Q with CTT or MTT were obtained by soaking preformed apo crystals in reservoir solution supplemented with 20 mM CTT or MTT for 1 h prior to data collection.

X-ray diffraction data were collected at beamline BL17U of the Shanghai Synchrotron Radiation Facility, Shanghai Institute of Applied Physics, Chinese Academy of Sciences. Data processing and scaling were performed using the HKL2000 software suite. All crystal structures were solved by molecular replacement using PHENIX Phaser-MR with the structure of *Aspergillus niger* 1,4-β-endoglucanase (PDB: 5I77; 64% sequence identity) as the search model. Subsequent model building and structural refinement were carried out using Coot and Refmac5, respectively. Data collection and refinement statistics are summarized in the supplemental material.

### Enzymatic characterization

The 3,5-dinitrosalicylic acid (DNS) method was used to determine CEL and MAN activities (31). Each reaction mixture contained 900 μL of substrate solution. The substrate was 1% (wt/vol) CMC-Na or 0.5% (wt/vol) LBG.

To determine the optimal pH, the substrates were dissolved in different buffer systems: glycine–HCl buffer for pH 1.0–3.0, citric acid–Na_2_HPO_4_ buffer for pH 3.0–8.0, and Tris-HCl buffer for pH 8.0–10.0. Enzyme activities were assayed at 37°C, and the pH yielding the highest activity was defined as the optimal pH. To determine the optimal temperature, enzyme activities were measured at 30°C–90°C (at 5°C intervals) under the optimal pH condition, and the temperature corresponding to the highest activity was defined as the optimal temperature.

Subsequently, 100 μL of appropriately diluted enzyme solution was added to the reaction mixture, and the reaction was incubated for 10 min under the corresponding pH and temperature conditions. After incubation, 1.5 mL of DNS reagent was added, and the mixture was boiled for 5 min to terminate the reaction and allow color development. The amount of reducing sugars released was quantified by measuring the absorbance at 540 nm. One unit of CEL or MAN activity was defined as the amount of enzyme required to release 1 μmol of reducing sugar per minute under the assay conditions.

Enzyme kinetic parameters were determined using substrate concentrations ranging from 1 to 10 mg/mL for CMC-Na and 1 to 7.5 mg/mL for LBG, under optimal assay conditions with a reaction time of 5 min. The values of *K*_m_ and *V*_max_ were calculated using nonlinear regression analysis in GraphPad Prism 9.0 (GraphPad Software Inc., San Diego, CA, USA). The turnover number (*k*_cat_) and catalytic efficiency (*k*_cat_/*K*_m_) were derived from the determined *K*_m_ and *V*_max_ values. All reactions were performed in triplicate.

## Data Availability

The atomic coordinates of the wild-type BsCel5B and the E142Q/E257Q mutant in complex with CTT and MTT have been deposited in the Protein Data Bank (PDB) under the accession numbers 8ZLG (WT), 8ZIK (E142Q/E257Q–CTT), and 8ZI5 (E142Q/E257Q–MTT). The data that support the findings of this study are available from the corresponding author upon reasonable request.

## SUPPLEMENTAL MATERIAL

Supplemental material aem.00417-26-s0001.docx Fig. S1; Tables S1 to S6.

ASM does not own the copyrights to Supplemental Material that may be linked to, or accessed through, an article. The authors have granted ASM a non-exclusive, world-wide license to publish the Supplemental Material files. Please contact the corresponding author directly for reuse.

## References

[1] Golder HM, Rossow HA, Lean IJ. 2019. Effects of in-feed enzymes on milk production and components, reproduction, and health in dairy cows. J Dairy Sci 102:8011–8026. doi:10.3168/jds.2019-16601 31279550

[2] Madadi M, Song GJ, Sun FB, Sun CH, Xia CL, Zhang EZ, Karimi K, Tu MB. 2022. Positive role of non-catalytic proteins on mitigating inhibitory effects of lignin and enhancing cellulase activity in enzymatic hydrolysis: application, mechanism, and prospective. Environ Res 215:114291. doi:10.1016/j.envres.2022.114291 36103929

[3] Kumar R, Wyman CE. 2014. Strong cellulase inhibition by Mannan polysaccharides in cellulose conversion to sugars. Biotechnol Bioeng 111:1341–1353. doi:10.1002/bit.25218 24522973

[4] Glasgow E, Vander Meulen K, Kuch N, Fox BG. 2021. Multifunctional cellulases are potent, versatile tools for a renewable bioeconomy. Curr Opin Biotechnol 67:141–148. doi:10.1016/j.copbio.2020.12.020 33550093 PMC8366578

[5] Paul M, Mohapatra S, Kumar Das Mohapatra P, Thatoi H. 2021. Microbial cellulases - an update towards its surface chemistry, genetic engineering and recovery for its biotechnological potential. Bioresour Technol 340:125710. doi:10.1016/j.biortech.2021.125710 34365301

[6] Wang K, Luo HY, Bai YG, Shi PJ, Huang HQ, Xue XL, Yao B. 2014. A thermophilic endo-1,4-β-glucanase from Talaromyces emersonii CBS394.64 with broad substrate specificity and great application potentials. Appl Microbiol Biotechnol 98:7051–7060. doi:10.1007/s00253-014-5680-0 24668246

[7] Zheng F, Vermaas JV, Zheng J, Wang Y, Tu T, Wang XY, Xie XM, Yao B, Beckham GT, Luo HY. 2019. Activity and thermostability of GH5 endoglucanase chimeras from mesophilic and thermophilic parents. Appl Environ Microbiol 85:e02079-18. doi:10.1128/AEM.02079-18 30552196 PMC6384118

[8] Payne CM, Knott BC, Mayes HB, Hansson H, Himmel ME, Sandgren M, Ståhlberg J, Beckham GT. 2015. Fungal cellulases. Chem Rev 115:1308–1448. doi:10.1021/cr500351c 25629559

[9] Vick JE, Schmidt DMZ, Gerlt JA. 2005. Evolutionary potential of (β/α)_8_-barrels: in vitro enhancement of a "new" reaction in the enolase superfamily. Biochemistry 44:11722–11729. doi:10.1021/bi050963g 16128573

[10] Varadarajan N, Gam J, Olsen MJ, Georgiou G, Iverson BL. 2005. Engineering of protease variants exhibiting high catalytic activity and exquisite substrate selectivity. Proc Natl Acad Sci USA 102:6855–6860. doi:10.1073/pnas.0500063102 15867160 PMC1100772

[11] Chang CW, Wehner D, Prabhu GRD, Moon E, Safferthal M, Bechtella L, Österlund N, Vos GM, Pagel K. 2025. Elucidating reactive sugar-intermediates by mass spectrometry. Commun Chem 8:67. doi:10.1038/s42004-025-01467-5 40055429 PMC11889121

[12] Pereira JH, Chen Z, McAndrew RP, Sapra R, Chhabra SR, Sale KL, Simmons BA, Adams PD. 2010. Biochemical characterization and crystal structure of endoglucanase Cel5A from the hyperthermophilic Thermotoga maritima. J Struct Biol 172:372–379. doi:10.1016/j.jsb.2010.06.018 20599513

[13] Wu T-H, Huang C-H, Ko T-P, Lai H-L, Ma Y, Chen C-C, Cheng Y-S, Liu J-R, Guo R-T. 2011. Diverse substrate recognition mechanism revealed by Thermotoga maritima Cel5A structures in complex with cellotetraose, cellobiose and mannotriose. Biochim Biophys Acta 1814:1832–1840. doi:10.1016/j.bbapap.2011.07.020 21839861

[14] Liang PH, Lin WL, Hsieh HY, Lin TY, Chen CH, Tewary SK, Lee HL, Yuan SF, Yang B, Yao JY, Ho MC. 2018. A flexible loop for mannan recognition and activity enhancement in a bifunctional glycoside hydrolase family 5. Biochim Biophys Acta Gen Subj 1862:513–521. doi:10.1016/j.bbagen.2017.11.004 29108954

[15] Mazurenko S, Prokop Z, Damborsky J. 2020. Machine learning in enzyme engineering. ACS Catal 10:1210–1223. doi:10.1021/acscatal.9b04321

[16] Kumagai Y, Yamashita K, Tagami T, Uraji M, Wan K, Okuyama M, Yao M, Kimura A, Hatanaka T. 2015. The loop structure of Actinomycete glycoside hydrolase family 5 mannanases governs substrate recognition. FEBS J 282:4001–4014. doi:10.1111/febs.13401 26257335

[17] Lv KM, Li XZ, Chen KQ, Wu B, He BF, Schenk G. 2024. Improving the catalytic efficiency of a GH5 processive endoglucanase by a combinatorial strategy using consensus mutagenesis and loop engineering. ACS Catal 14:6856–6867. doi:10.1021/acscatal.4c01083

[18] Yan JJ, Liu WD, Li YJ, Lai HL, Zheng YY, Huang JW, Chen CC, Chen Y, Jin J, Li HZ, Guo RT. 2016. Functional and structural analysis of Pichia pastoris-expressed Aspergillus niger 1,4-β-endoglucanase. Biochem Biophys Res Commun 475:8–12. doi:10.1016/j.bbrc.2016.05.012 27154222

[19] Chen ZW, Friedland GD, Pereira JH, Reveco SA, Chan R, Park JI, Thelen MP, Adams PD, Arkin AP, Keasling JD, Blanch HW, Simmons BA, Sale KL, Chivian D, Chhabra SR. 2012. Tracing determinants of dual substrate specificity in glycoside hydrolase family 5. J Biol Chem 287:25335–25343. doi:10.1074/jbc.M112.362640 22645145 PMC3408205

[20] Newton MS, Guo XH, Söderholm A, Näsvall J, Lundström P, Andersson DI, Selmer M, Patrick WM. 2017. Structural and functional innovations in the real-time evolution of new (βα)_8_ barrel enzymes. Proc Natl Acad Sci USA 114:4727–4732. doi:10.1073/pnas.1618552114 28416687 PMC5422803

[21] Pinney MM, Mokhtari DA, Akiva E, Yabukarski F, Sanchez DM, Liang RB, Doukov T, Martinez TJ, Babbitt PC, Herschlag D. 2021. Parallel molecular mechanisms for enzyme temperature adaptation. Science 371:eaay2784. doi:10.1126/science.aay2784 33674467 PMC13050526

[22] Hoffmeister D, Yang J, Liu L, Thorson JS. 2003. Creation of the first anomeric D/L-sugar kinase by means of directed evolution. Proc Natl Acad Sci USA 100:13184–13189. doi:10.1073/pnas.100.23.13184 14612558 PMC263743

[23] Fröhlich C, Bunzel HA, Buda K, Mulholland AJ, van der Kamp MW, Johnsen PJ, Leiros H-KS, Tokuriki N. 2024. Epistasis arises from shifting the rate-limiting step during enzyme evolution of a β-lactamase. Nat Catal 7:499–509. doi:10.1038/s41929-024-01117-4 38828429 PMC11136654

[24] Buda K, Miton CM, Tokuriki N. 2023. Pervasive epistasis exposes intramolecular networks in adaptive enzyme evolution. Nat Commun 14:8508. doi:10.1038/s41467-023-44333-5 38129396 PMC10739712

[25] Acevedo-Rocha CG, Li AT, D’Amore L, Hoebenreich S, Sanchis J, Lubrano P, Ferla MP, Garcia-Borràs M, Osuna S, Reetz MT. 2021. Pervasive cooperative mutational effects on multiple catalytic enzyme traits emerge via long-range conformational dynamics. Nat Commun 12:1621. doi:10.1038/s41467-021-21833-w 33712579 PMC7955134

[26] Osuna S. 2021. The challenge of predicting distal active site mutations in computational enzyme design. WIREs Comput Mol Sci 11:e1502. doi:10.1002/wcms.1502

[27] Mistry J, Chuguransky S, Williams L, Qureshi M, Salazar GA, Sonnhammer ELL, Tosatto SCE, Paladin L, Raj S, Richardson LJ, Finn RD, Bateman A. 2021. Pfam: the protein families database in 2021. Nucleic Acids Res 49:D412–D419. doi:10.1093/nar/gkaa913 33125078 PMC7779014

[28] Katoh K, Frith MC. 2012. Adding unaligned sequences into an existing alignment using MAFFT and LAST. Bioinformatics 28:3144–3146. doi:10.1093/bioinformatics/bts578 23023983 PMC3516148

[29] Price MN, Dehal PS, Arkin AP. 2010. FastTree 2--approximately maximum-likelihood trees for large alignments. PLoS One 5:e9490. doi:10.1371/journal.pone.0009490 20224823 PMC2835736

[30] Zheng J, Liu H, Qin X, Yang K, Tian J, Wang X, Wang Y, Wang Y, Yao B, Luo H, Huang H. 2022. Identification and mutation analysis of nonconserved residues on the TIM-barrel surface of GH5_5 cellulases for catalytic efficiency and stability improvement. Appl Environ Microbiol 88. doi:10.1128/aem.01046-22 PMC9469711 36000858

[31] Gu X, Lu H, Chen W, Meng X. 2021. Characterization of a novel thermophilic mannanase and synergistic hydrolysis of galactomannan combined with swollenin. Catalysts 11:254. doi:10.3390/catal11020254

