# Supplementary material for: Deciphering functional diversity and structural determinants of substrate specificity in fungal glycoside hydrolase family 5_5 cellulases
Source: Appl Environ Microbiol. 2026 Apr 21;92(5):e00417-26. doi: 10.1128/aem.00417-26 (PMC13188850; doi:10.1128/aem.00417-26)
Supplement: Supplemental material — Fig. S1; Tables S1 to S6. [file aem.00417-26-s0001.docx]

**Supplemental Material**

**Deciphering functional diversity and structural determinants of substrate specificity in fungal glycoside hydrolase family 5_5 cellulases**


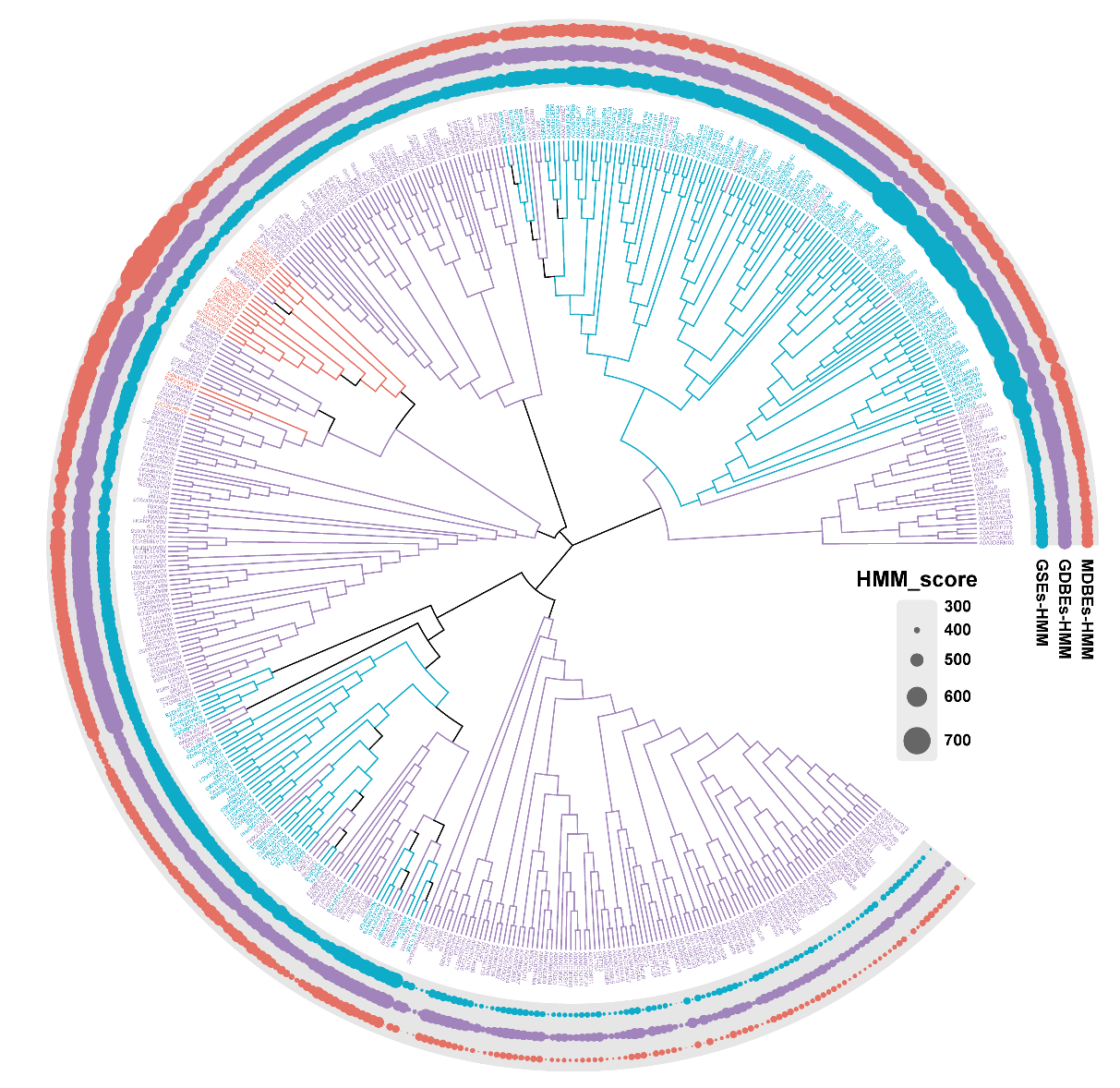


**FIG S1** Phylogenetic analysis of multifunctional cellulases mined from the database.

**Table S1** The 25 GH5_5 fungal cellulases used in this study.

| No. | Enzyme | Accession number | Species (Strain) |
| --- | --- | --- | --- |
| 1 | *Ao*Cel5A | B8MW97 | *Aspergillus oryzae* |
| 2 | *Ae*Cel5A | A0A319DKY6 | *Aspergillus ellipticus* CBS 707.79 |
| 3 | *An*Cel5A | O74706 | *Aspergillus niger* |
| 4 | *Ai*Cel5A | A0A2V5I303 | *Aspergillus indologenus* CBS 114.80 |
| 5 | *Ta*Cel5A | Q8TG26 | *Thermoascus aurantiacus* |
| 6 | *Ac*Cel5A | A0A0U5CPV4 | *Aspergillus calidoustus* |
| 7 | *Tl*Cel5A | A0A3G5ECP2 | *Talaromyces leycettanus* JCM12802 |
| 8 | *Te*Egl5A | Q8WZD7 | *Talaromyces emersonii* |
| 9 | *Np*Cel5A | R1GRI8 | *Neofusicoccum parvum* UCRNP2 |
| 10 | *Mp*Cel5A | Q12638 | *Macrophomina phaseolina* |
| 11 | *So*Cel5A | A0A1W6R2C1 | *Stegonsporium opalus* |
| 12 | *Il*Cel5A | Q5W7K4 | *Irpex lacteus* |
| 13 | *Ba*Cel5A | M1G8Y4 | *Bispora antennata* |
| 14 | *Hw*Cel5A | A0A1Z5STI4 | *Hortaea werneckii* |
| 15 | *Tl*Cel5A | A0A3G5ECP2 | *Talaromyces leycettanus* JCM12802 |
| 16 | *Dc*Cel5A | A0A1J9QK41 | *Diplodia corticola* |
| 17 | *Am*Cel5A | UPI00080550B5 | *Aureobasidium melanogenum* |
| 18 | *Ap*Cel5B | OBW63759 | *Aureobasidium pullulans* |
| 19 | *Ap*Cel5A | A0A4S9KXJ9 | *Aureobasidium pullulans* |
| 20 | *An*Cel5B | A0A074WGE8 | *Aureobasidium namibiae* |
| 21 | *As*Cel5 | A0A074YK40 | *Aureobasidium subglaciales* |
| 22 | *Pf*Cel5A | N1Q8M5 | *Pseudocercospora fijiensis* |
| 23 | *Ds*Cel5A | N1PH22 | *Dothistroma septosporum NZE* |
| 24 | *Bs*Cel5B | A0A150VE32 | *Bispora* sp. MEY-1 |
| 25 | *Gs*Cel5A | A0A8E2EQ63 | *Glonium stellatum* |

**Table S2** Optimal reaction conditions of 25 cellulases on CMC-Na and LBG.

| Enzymes | Types | CMC-Na | | LBG | | Data source |
| --- | --- | --- | --- | --- | --- | --- |
|  |  | Temperature (℃) | pH | Temperature (℃) | pH |  |
| *Ao*Cel5A | GSE | 60 | 4.0 | 60 | 4.0 | Experiment |
| *Ae*Cel5A | GSE | 70 | 4.0 | 70 | 4.0 | Experiment |
| *An*Cel5A | GSE | 75 | 4.0 | 75 | 4.0 | Experiment |
| *Ai*Cel5A | GSE | 75 | 4.0 | 75 | 4.0 | Experiment |
| *Ta*Cel5A | GSE | 70 | 3-4.5 |  |  | Reference(1) |
| *Ac*Cel5A | GSE | 60 | 4.0 | 60 | 4.0 | Experiment |
| *Tl*Cel5A | GDBE | 70 | 4.0 | 70 | 4.0 | Experiment |
| *Te*Egl5A | GDBE | 90 | 4.5 | 90 | 4.5 | Experiment |
| *Np*Cel5A | GDBE | 60 | 5.0 | 60 | 5.0 | Experiment |
| *Mp*Cel5A | GDBE | 60 | 5.0 | 60 | 5.0 | Experiment |
| *So*Cel5A | GDBE | 60 | 5.0 | 60 | 5.0 | Experiment |
| *IlCel5A* | GDBE | 50 | 5.0 |  |  | Reference(2) |
| *Ba*Cel5A | GDBE | 50 | 4.0 | 50 | 4.0 | Experiment |
| *Hw*Cel5A | GDBE | 75 | 5.0 | 75 | 5.0 | Experiment |
| *Tv*Cel5A | GDBE | 80 | 4.0 | 80 | 4.0 | Experiment |
| *Dc*Cel5A | GDBE | 60 | 5.0 | 60 | 5.0 | Experiment |
| *Am*Cel5A | MDBE | 65 | 5.0 | 65 | 5.0 | Experiment |
| *Ap*Cel5B | MDBE | 60 | 5.0 | 60 | 5.0 | Experiment |
| *Ap*Cel5A | MDBE | 60 | 4.0 | 60 | 4.0 | Experiment |
| *An*Cel5B | MDBE | 60 | 5.0 | 60 | 5.0 | Experiment |
| *As*Cel5 | MDBE | 65 | 5.0 | 65 | 5.0 | Experiment |
| *Pf*Cel5A | MDBE | 65 | 4.5 | 65 | 4.5 | Experiment |
| *Ds*Cel5A | MDBE | 60 | 4.0 | 60 | 4.0 | Experiment |
| *Bs*Cel5B | MDBE | 80 | 4.0 | 80 | 4.0 | Experiment |
| *Gs*Cel5A | MDBE | 55 | 4.5 | 55 | 4.5 | Experiment |

**Table S3** Leave-one-out (LOO) cross-validation of HMM models for enzyme classification.

| Enzymes | HMM score | | | | Δscore  (LOO – Next Best) |
| --- | --- | --- | --- | --- | --- |
|  | GSE | GDBE | MDBE | LOO |  |
| *Ao*Cel5A | 648.2 | 541.2 | 495.5 | 617.2 | 76 |
| *Ae*Cel5A | 645.2 | 527.2 | 478.6 | 623 | 95.8 |
| *An*Cel5A | 642.2 | 533.4 | 488.9 | 615.8 | 82.4 |
| *Ai*Cel5A | 637.5 | 528.8 | 483.5 | 609.3 | 80.5 |
| *Ta*Cel5A | 636.6 | 593.7 | 519.9 | 563.8 | -29.9 |
| *Ac*Cel5A | 619.4 | 533.7 | 478.8 | 570.6 | 36.9 |
| *Tl*Cel5A | 555.8 | 622.2 | 500 | 546.3 | -9.5 |
| *Te*Egl5A | 550.4 | 601.6 | 496.2 | 564.5 | 14.1 |
| *Np*Cel5A | 493.6 | 588.6 | 536.8 | 563.8 | 27 |
| *Mp*Cel5A | 495.1 | 581.7 | 522.9 | 561.7 | 38.8 |
| *So*Cel5A | 457 | 572.3 | 484.8 | 524.1 | 39.3 |
| *Il*Cel5A | 397.9 | 570 | 405.2 | 448.5 | 43.3 |
| *Ba*Cel5A | 455.2 | 567.5 | 453 | 511.2 | 56 |
| *Hw*Cel5A | 437.4 | 565 | 546.1 | 511 | -35.1 |
| *Tv*Cel5A | 532.9 | 562.4 | 483.7 | 535.1 | 2.2 |
| *Dc*Cel5A | 467.5 | 560.8 | 496.7 | 522.6 | 25.9 |
| *Am*Cel5A | 462.2 | 521.7 | 657.9 | 650.9 | 129.2 |
| *Ap*Cel5B | 462.2 | 521.7 | 657.9 | 650.9 | 129.2 |
| *Ap*Cel5A | 474.1 | 531.7 | 654.3 | 647.7 | 116 |
| *An*Cel5B | 460.1 | 520.3 | 649.2 | 639.3 | 119 |
| *As*Cel5 | 466.9 | 523.6 | 648.3 | 635.7 | 112.1 |
| *Pf*Cel5A | 484.9 | 532 | 629.8 | 588.6 | 56.6 |
| *Ds*Cel5A | 463.3 | 532.2 | 621.4 | 578.8 | 46.6 |
| *Bs*Cel5B | 473.4 | 523.7 | 613.2 | 553.4 | 29.7 |
| *Gs*Cel5A | 511.3 | 574 | 591.8 | 534.7 | -39.3 |

ΔScore indicates the discrimination gap between the LOO score and the highest alternative HMM score for each enzyme sequence.

**Table S4** Frequency analysis of promiscuity-associated residues across subtypes.

| *Bs*Cel5B position | GSE | | GDBE | | MDBE | |
| --- | --- | --- | --- | --- | --- | --- |
|  | Consensus residue | Frequency (%) | Consensus residue | Frequency (%) | Consensus residue | Frequency (%) |
| 60 | P | 46.5 | P | 51.0 | N | 88.9 |
| 61 | F | 34.4 | F | 43.8 | L | 72.3 |
| 89 | A | 29.2 | Y | 74.4 | Y | 50.0 |
| 93 | S | 31.3 | K | 75.1 | K | 44.4 |
| 98 | V | 70.1 | V | 39.2 | M | 38.9 |
| 100 | D | 100 | D | 58.4 | V | 44.4 |
| 148 | Q | 98.1 | Q | 35.3 | G | 55.5 |
| 152 | L | 91.1 | F | 37.0 | A | 89.9 |
| 173 | F | 94.3 | F | 35.6 | T | 61.1 |
| 178 | S | 74,1 | S | 82.3 | A | 66.7 |
| 179 | W | 67.7 | W | 69.4 | Y | 66.7 |
| 187 | D | 34.1 | S | 62.8 | T | 83.3 |
| 189 | V | 54.8 | S | 81.5 | Q | 38.9 |
| 190 | N | 100.0 | G | 82.6 | G | 76.5 |
| 201 | K | 77.2 | G | 33.2 | G | 94.4 |
| 208 | D | 62.6 | N | 62.7 | N | 55.6 |
| 251 | K | 32.9 | K | 57.2 | I | 38.9 |
| 266 | K | 32.5 | I | 54.6 | E | 70.6 |
| 275 | D | 54.5 | C | 31.5 | A | 41.2 |
| 278 | S | 22.9 | Q | 41.0 | A | 29.4 |
| 303 | F | 34.6 | I | 46.9 | V | 56.3 |
| 313 | Y | 40.1 | G | 14.8 | T | 81.3 |
| 315 | L | 51.0 | L | 68.3 | V | 37.5 |
| 317 | L | 37.6 | A | 35.1 | I | 43.8 |
| 319 | K | 49.0 | K | 31.9 | A | 37.5 |
| 320 | P | 27.4 | P | 38.9 | S | 31.3 |

**Table S5** Cα distance to catalytic center for key residues in *Bs*Cel5B.

| *Bs*Cel5B | Cα distance to catalytic center (Å) | |
| --- | --- | --- |
|  | E142 | E257 |
| N60 | 11.91 | 11.61 |
| L61 | 13.09 | 15.09 |
| Y89 | 26.14 | 21.91 |
| K93 | 28.62 | 22.76 |
| M98 | 14.91 | 12.57 |
| T100 | 10.45 | 12.03 |
| G148 | 12.45 | 20.75 |
| A152 | 12.63 | 20.01 |
| N173 | 11.73 | 12.54 |
| S178 | 7.01 | 13.94 |
| Y179 | 3.99 | 10.56 |
| T187 | 16.00 | 19.39 |
| E189 | 14.19 | 20.82 |
| G190 | 12.70 | 20.88 |
| G201 | 17.18 | 20.69 |
| D208 | 22.57 | 22.98 |
| K251 | 19.26 | 18.55 |
| C266 | 22.95 | 14.95 |
| D275 | 24.61 | 15.88 |
| D278 | 24.08 | 16.05 |
| V303 | 21.41 | 13.05 |
| T313 | 26.83 | 17.23 |
| D315 | 24.79 | 14.90 |
| I317 | 25.68 | 15.42 |
| L319 | 26.19 | 16.19 |
| E320 | 27.71 | 17.67 |

**Table S6** X-ray data collection and structure refinement statistics.

| PDB ID | WT | E142Q/E257Q-CTT | E142Q/E257Q-MTT |
| --- | --- | --- | --- |
|  | 8ZLG | 8ZIK | 8ZI5 |
| **Data co1lection** |  |  |  |
| Space group | *I*41 | *P*2_1_2_1_2_1_ | *P*2_1_2_1_2_1_ |
| *a, b, c* (Å) | 115.10,115.10, 107.71 | 72.32,94.99,179.76 | 69.72, 88.59, 175.74 |
| *α，β，γ* (°) | 90,90,90 | 90,90,90 | 90,90,90 |
| Unique reflections | 27240 | 92829 | 110688 |
| Resolution (Å) | 40.69 - 2.25  (2.335 - 2.25) | 29.86 - 1.89  (1.92 - 1.89) | 18.51 - 1.73  (1.79 - 1.73) |
| Completeness (%) | 80.67 (82.17) | 90.15 (64.88) | 97.15 (98.14) |
| Average I/σ | 6.30 | 3.34 | 3.32 |
| CC 1/2 | 0.961 | 0.996 | 0.964 |
| CC * | 0.990 | 0.920 | 0.991 |
| **Refinement** |  |  |  |
| Rwork (95 % of data) | 0.183 (0.212) | 0.199 | 0.151 |
| Rfree (5 % of data) | 0.248 (0.326) | 0.229 | 0.196 |
| r.m.s.d. bonds (Å) | 0.008 | 0.003 | 0.007 |
| r.m.s.d. angles (º) | 0.972 | 0.072 | 0.987 |
| Ramachandran outliers (%) | 0.00 | 0.00 | 0.00 |

**REFERENCES**

1. Lo Leggio L, Larsen S. 2002. The 1.62 Å structure of *Thermoascus aurantiacus* endoglucanase: completing the structural picture of subfamilies in glycoside hydrolase family 5. FEBS Lett 523:103-108.

2. Toda H, Takada S, Oda M, Amano Y, Kanda T, Okazaki M, Shimosaka M. 2005. Gene cloning of an endoglucanase from the basidiomycete *Irpex lacteus* and its cDNA expression in *Saccharomyces cerevisiae*. Biosci, Biotechnol, Biochem 69:1262-1269.
